# Emerging roles of tripartite motif family proteins (TRIMs) in breast cancer

**DOI:** 10.1002/cam4.7472

**Published:** 2024-07-17

**Authors:** Jianing Cao, Mengdi Yang, Duancheng Guo, Zhonghua Tao, Xichun Hu

**Affiliations:** ^1^ Department of Breast and Urologic Medical Oncology Fudan University Shanghai Cancer Center Shanghai China; ^2^ Department of Oncology Shanghai Medical College, Fudan University Shanghai China

**Keywords:** breast cancer, E3 ubiquitin ligase, TRIM proteins, ubiquitination

## Abstract

Breast cancer (BC) is the most common malignant tumor worldwide. Despite enormous progress made in the past decades, the underlying mechanisms of BC remain further illustrated. Recently, TRIM family proteins proved to be engaged in BC progression through regulating various aspects. Here we reviewed the structures and basic functions of TRIM family members and first classified them into three groups according to canonical polyubiquitination forms that they could mediate: K48‐ only, K63‐ only, and both K48‐ and K63‐linked ubiquitination. Afterwards, we focused on the specific biological functions and mechanisms of TRIMs in BCs, including tumorigenesis and invasiveness, drug sensitivity, tumor immune microenvironment (TIME), cell cycle, and metabolic reprogramming. We also explored the potential of TRIMs as novel biomarkers for predicting prognosis and future therapeutic targets in BC.

## 1 INTRODUCTION

Worldwide, breast cancer (BC) has been the most frequently newly diagnosed type of cancer and emerged as an increasingly heavy disease burden.^1^ During the past decades, the survival of BC, especially those at early stage, has largely improved, which attributed to cutting‐edge research and advances in surgery, radiotherapy, chemotherapy, and endocrine therapy. Despite major improvements, owing to the lack of effective targeted therapy for late‐stage patients, BC remains the leading cause of cancer death.^1^ Therefore, further deep molecular and cellular profiling is needed to better understand its progression and help discover individualized precision medicine.

The tripartite motif (TRIM) family proteins are involved in a variety of biological processes, including cell proliferation,^2^, ^3^ cell cycle,^4^ stress response,^5^ and pluripotency.^6^ Thus, abnormal expressions of TRIMs are usually associated with different pathological conditions.^7^, ^8^ This review conducted a comprehensive and intensive investigation of the TRIMs' functions in BC, including tumorigenesis, proliferation, apoptosis, metastasis and invasion, drug resistance, tumor immune microenvironment (TIME) and immune response, cell division, and metabolic reprogramming. Besides, we also discussed the diagnostic, prognostic, and therapeutic values of TRIM proteins, which might be the focus of research for years to come.

## 2 STRUCTURE AND BASIC FUNCTIONS OF TRIMs

Nearly 80 proteins are now considered to be members of the TRIM family, with the most distinctive feature of the highly conserved RBCC domains at the N‐terminal, which comprised of three typical domains: the RING finger (R), one or two B‐boxes (B1/B2), and coiled‐coil (CC).^7^ The CC region is responsible for mediating the hetero‐ or homo‐dimerization of TRIMs, while the B1/B2 domain is identified important for protein‐protein interactions. The RING finger domain mediates proteins conjugation with ubiquitin, or ubiquitin‐like molecule (ISG15 or SUMO).^9^, ^10^

As the catalytic site, this RING finger domain characterizes many E3 ubiquitin ligases. It is well established that the ubiquitin contains seven lysine (Lys) residues amenable of ubiquitination: K6, K11, K27, K29, K33, K48, and K63, and thus could produce seven kinds of ubiquitination. K48‐ and K63‐linked ubiquitination are the most studied cases. K48‐linked polyubiquitination generally is associated with the degradation of target proteins by the 26S proteasome system, whereas K63‐linked is involved in those nonproteolytic cellular functions, such as protein stabilization and activation,^11^, ^12^ DNA repair,^13^ and stress responses.^14^, ^15^ Here in Table 1, we summed up the roles of TRIM family proteins through these two predominant polyubiquitination forms, and accordingly categorized them into three groups: K48‐ only, K63‐ only, and those could undergo both K48‐ and K63‐linked ubiquitination. And in Table 2, we also provided a concise summary of recent advances in the other types of chains assembled through K6, K11, K27, K29, and K33 residues, which are less covered in the literature. Among the non‐canonical protein ubiquitination mediated by TRIM family members, K27‐linked polyubiquitination is the most frequently reported one and found heavily implicated in the regulation of innate immunity.

**TABLE 1:** TRIM family proteins categorized by the capacity to mediate K48 or K63 ubiquitination.

K48‐ only				K63‐ only				Both K48‐ and K63‐			
Subfamily member	Ubiquitinated molecules	Functions	References	Subfamily member	Ubiquitinated molecules	Functions	References	Subfamily member	Ubiquitinated molecules	Functions	References
TRIM6	IKKε	Antiviral response	^16^	TRIM8	TAK1	NF‐κB signaling	^17^	TRIM1	HIF1α/LRRK2	CRC progression/LRRK2 proteasomal degradation	18, 19
TRIM11	PHLPP1/KDM5C	Hepatocellular carcinogenesis/Oncogenic enhancer regulation	20, 21	TRIM13	NSP6	NF‐κB signaling	^22^	TRIM3	p53/Beclin 1/TLR3	Tumor proliferation/Autophagy/ Innate antiviral response and Cytokine storm	23, 24, 25, 26
TRIM14	Viral Nucleoprotein/Viral NS5A	IAV/HCV replication	27, 28	TRIM15	ERK1/2/LASP1	Melanoma development/Sensitivity to TKIs in HCC	29, 30	TRIM4	SET/RIG‐I	ER‐α expression/Antiviral response	31, 32
TRIM17	RBM38	Cisplatin resistance in NSCLC	^33^	TRIM18	PPM1A	Antiviral response	^34^	TRIM5	TAK1/NS2B/3	Anti‐HIV infection/Anti‐flaviviral infection	35, 36
TRIM27	ULK1/NOD2	Autophagy/NOD2‐mediated pro‐inflammatory responses	37, 38	TRIM24	TRAF3	Antiviral response	^39^	TRIM7	MAVS/MITA/NCOA4/RACO‐1/ATG7/ZIKV E protein/Src	Innate immune response/Ferritinophagy and ferroptosis/Lung carcinogenesis/Autophagy/ZIKV attachment and pathogenesis/HCC progression	40, 41, 42, 43, 44, 45, 46
TRIM29	MITA/IGF2BP1/NLRC4	Immune response/PD‐L1 expression in GC/NLRC4 inflammasome related cerebral injury	47, 48, 49, 50	TRIM32	MITA/ULK1	Antiviral response/Autophagy	51, 52	TRIM16	NLRP3/NRF2	Pyroptosis and inflammation/Expression of Antioxidant Genes	53, 54
TRIM30	Sox17/MITA	PTC cell proliferation and invasion/ Intracellular DNA and DNA virus‐triggered response	55, 56	TRIM33	Annexin A2	NF‐κB induced skin inflammation in psoriasis	^57^	TRIM21	TIF1γ/VDAC2/PEPCK1 & FASN/ATG5/DDX41/β‐catenin/p62/FSP1/CLASPIN/G3BP1/MST2/Keratin 17	Glioma progression/Irradiation‐induced mitochondrial DNA release/Metabolic disorders/Autophagy/Innate immune response/Antioxidant response/Ferroptosis/CHK1 activation/Stress granules formation/EMT of CRC/STAT3 activation	11, 12, 58, 59, 60, 61, 62, 63, 64, 65, 66, 67, 68
TRIM36	FOXA2	Ferroptosis in CRC	^69^	TRIM34	ZBP1	Influenza virus‐activated programmed cell death	^70^	TRIM22	Raf‐1/RIG‐I/IKKγ	Proliferation of GBM	71, 72, 73
TRIM38	TRIF/NAP1/TRAF6/MITA	TLR3/4‐mediated innate immune and inflammatory response/Replication of vesicular stomatitis virus/Immune tolerance at the maternal‐fetal interface	74, 75, 76, 77	TRIM45	p53/Atg5	Progression of GBM/Microglia pyroptosis	78, 79	TRIM23	ICP0/YFV NS5 protein	Virus replication/IFN‐I signaling	80, 81
TRIM39	PRDX3	Renal fibrosis	^82^	TRIM54	Filamin C	Progression of GC	^83^	TRIM25	RIP3/PTEN/RIG‐I/TRAF2	Cell necrosis/AKT/mTOR signaling in NSCLC/Innate immune responses/NF‐κB signaling	84, 85, 86, 87
TRIM40	MDA5 and RIG‐I (both K27‐ and K48‐linked polyubiquitination)	Antiviral immune response	^88^	TRIM56	ERα/MITA	ERα protein stability/Innate immune responses to intracellular double‐stranded DNA	89, 90	TRIM26	PBX1/IRF3/GPX4	NSCLC survival/Antiviral response/Ferroptosis	91, 92, 93
TRIM44	MAVS	Antiviral response	^94^	TRIM67	IκBα	NF‐κB activity after cerebral ischemia	^95^	TRIM28	MAVS/RIPK1/TBK1	RLR signaling/Anti‐PD‐1 resistance in NSCLC/PD‐L1 transcription in GC	96, 97, 98
TRIM46	TBK1	Influenza A H7N9 virus infection	^99^	TRIM68	MOAP1	NSCLC development	^100^	TRIM31	MAP3K7/TSC1/2/NLRP3 inflammasome/p53/MAVS	TGF‐β1 signaling/Protection against NASH/HCC progression/NLRP3 inflammasome activity/Progression of BC/Innate antiviral response	101, 102, 103, 104, 105, 106
TRIM48	PRMT1	ASK1 activation and cell death	^107^					TRIM35	LSD1/E1A/TRAF3/IAV PB2/IRF7/PKM2	Antitumor immunity in NSCLC/Adenovirus replication/Antiviral response/IFN‐I production/Heart failure	108, 109, 110, 111, 112
TRIM69	PRKCD	Anoikis resistance and metastasis of GC	^113^					TRIM37	p53/AP‐2γ/TRAF2/TRAF6	Progression of HCC/BC/NSCLC/Hepatic ischemia/reperfusion injury	114, 115, 116, 117
TRIM72	RAC1	HCC progression	^118^					TRIM41	ORF2p/BCL10	Preserve genome integrity/Innate antiviral response	119, 120
								TRIM47	NF90/TRAF2	Antiviral innate immunity/Inflammatory response in endothelial cells	121, 122
								TRIM50	Src/SNAIL/JUP	Progression of OC/EMT of HCC/MYC signaling and GC progression	123, 124, 125
								TRIM59	TRAF6/WASP	Autophagy in NSCLC/Embryo development from blastocyst stage to gastrula	126, 127
								TRIM65	VCAM‐1/MDA5	Endothelial inflammatory response/Antiviral innate immunity	128, 129

*Note*: Red, suggested as a promoting role. Green, suggested as a suppressing role.

Abbreviations: AP‐2γ, activator protein 2 gamma; ATG5, autophagy‐related gene 5; ATG7, autophagy related genes 7; BCL10, B cell lymphoma 10; CRC, colorectal cancer; EMT, epithelial‐to‐mesenchymal transition; ER, estrogen receptor; ERK, extracellular‐signal‐regulated kinase; FASN, fatty acid synthase; FOXA2, forkhead box transcription factor A2; FSP1, ferroptosis suppressor protein 1; GC, gastric cancer; G3BP1, G3BP stress granule assembly factor 1; GBM, glioblastoma; HCC, hepatocellular carcinoma; HCV, hepatitis C virus; HRD, hypertensive renal disease; IAV, influenza A virus; LRRK2, leucine‐rich repeat kinase 2; LSD1, lysine‐specific histone demethylase 1A; MAP3K7, mitogen‐activated protein kinase kinase kinase 7; MAVS, mitochondrial antiviral signaling protein (also known as virus‐induced signal adaptor, VISA); MITA, mediator of IRF3 activation (also known as stimulator of interferon genes, STING); MOAP1, modulator of apoptosis‐1; NAP1, NF‐κB‐activating kinase‐associated protein 1; NASH, nonalcoholic steatohepatitis; NCOA4, nuclear receptor coactivator 4; NF90, nuclear factor 90; NLRC4, NLR family CARD domain containing protein 4; NLRP3, nucleotide‐binding oligomerization domain‐like receptor family pyrin domain containing 3; NRF2, nuclear factor erythroid 2‐related factor 2; NSCLC, non‐small cell lung cancer; NSP6, nonstructural protein 6; OC, ovarian cancer; PEPCK, phosphoenolpyruvate carboxykinase 1; PHLPP1, pleckstrin homology domain leucine‐rich repeats protein phosphatase 1; PPM1A, protein phosphatase 1A; PRMT1, protein arginine methyltransferase 1; PTC, papillary thyroid cancer; RAC1, ras‐related C3 botulinum toxin substrate 1; RACO‐1, RING domain AP‐1 co‐activator 1; RIG‐I, retinoic acid‐inducible gene‐I; RLR, RIG‐I‐like receptors (including melanoma differentiation‐associated gene 5 (MDA5) and RIG‐I); RIP3, receptor interacting protein kinase 3; RIPK1, receptor‐interacting protein kinase 1; SET, SE translocation; TAK1, transforming growth factor‐β‐activated kinase 1; TAB1, TAK1‐binding protein 1; TBK1, TANK‐binding kinase 1; TKI, tyrosine kinase inhibitor; TRAF, TNF receptor associated factor; TRIF, TIR domain–containing adapter‐inducing IFN‐β; TSC, tuberous sclerosis complex; VCAM‐1, vascular cell adhesion molecule 1; VDAC2, mitochondrial voltage‐dependent anion‐selective channel protein 2; YFV, yellow fever virus; WASP, Wiskott–Aldrich syndrome protein; ZBP1, Z‐DNA‐binding protein 1.

**TABLE 2:** TRIM family proteins categorized by the capacity to mediate noncanonical protein ubiquitination.

K6‐ and K33‐				K11‐				K27‐			
Subfamily member	Ubiquitinated molecules	Functions	References	Subfamily member	Ubiquitinated molecules	Functions	References	Subfamily member	Ubiquitinated molecules	Functions	References
TRIM8	TRIF	Innate immune response	^130^	TRIM3	SCL7A11	NSCLC development	^131^	TRIM10	MITA (both K27‐ and K29‐ linked polyubiquitination)	Innate immune response	^132^
K29‐				TRIM21	LPP	Lymphatic metastasis of bladder cancer	^133^	TRIM21	MAVS	Innate antiviral response	^134^
TRIM13	TRAF6	NF‐κB signaling	^135^	TRIM26	TAB1	Inflammatory innate immune response	^136^	TRIM23	NEMO/TRIM23	Antiviral response/Autophagy	137, 138
								TRIM26	TRIM26	Antiviral response	^139^
								TRIM31	SYK	Antifungal immunity	^140^

*Note*: Red, suggested as a promoting role. Green, suggested as a suppressing role.

Abbreviations: LPP, lipoma preferred partner; NEMO, NF‐kappaB essential modulator; SYK, spleen tyrosine kinase.

**FIGURE 1:**
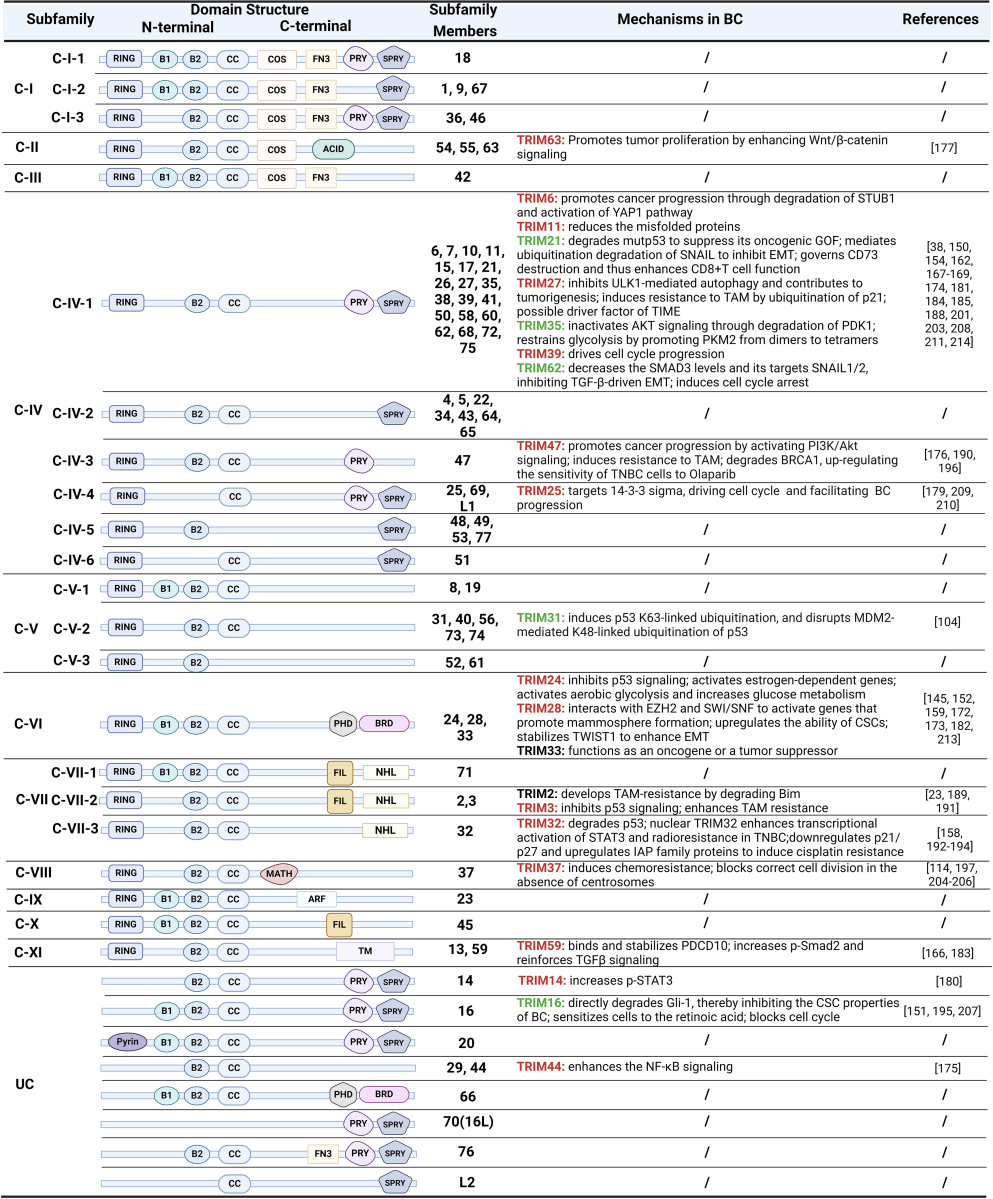
Illustration of TRIM proteins domain structure, classification, and essential mechanisms in BC. Red, suggested as a promoting role. Green, suggested as a suppressing role. Black, suggested unknown.

The C‐terminal of the TRIM family is a highly variable carboxyl‐terminal domain, which includes: COS domain, fibronectin type III repeat (FNIII), PRY domain, SPRY domain, acid‐rich region (ACID), PHD domain, bromodomain (BRD), filamin‐type IG domain (FIL), NHL domain, Meprin and TRAF‐homology domain (MATH), ADP‐ribosylation factor family domain (ARF), and transmembrane region (TM).^141^, ^142^ The structural diversity of C‐terminal is a predictor of the functional diversity of the TRIM family. Approximately 2/3 of TRIM proteins have a common SPRY domain, including the B30.2 domain (also known as the PRY‐SPRY domain, which contains a PRY extension at the N‐terminus) and the “SPRY‐only” subfamilies, which exhibits a crucial role in the innate immune responses, and subcellular localization.^143^ In addition, TRIM23 of the C‐XI subfamily contains an ARF domain that participates in virus‐induced autophagy.^138^ The C‐VI subfamily members with PHD‐BRD feature are epigenome readers that control gene expression by recruiting multiprotein complexes of transcription factors and chromatin regulators.^144^, ^145^ Depending on the C‐terminal, TRIM proteins can be divided into 11 subfamilies and an unclassified (UC) group (Figure 1). The eight proteins concluded in the UC subfamily do not contain the RING finger domain and hence do not possess ubiquitin ligase activity. Nevertheless, they are still classified to be members of the TRIM family, considering their conserved B‐boxes and CC domains.^146^

## 3 THE ROLES OF TRIMs IN BC

### 3.1 Tumorgenesis

Protein quality control (PQC) is indispensable for eukaryotic cells to remove misfolded proteins and maintain cellular homeostasis. The TRIM family members have been established a critical role in PQC.^147^, ^148^, ^149^ A recent study in a human mammary epithelial cells (HMECs) transformation model indicated that the proteasome activity, especially TRIM proteins is usually upregulated during oncogenic transformation, implying its necessity for the initiation of malignant phenotypes. When researchers proceeded with more detailed analyses of TRIM11 in the study, they found forced expression of TRIM11 could reduce the inclusion bodies formed by the exogenous misfolded proteins Atxn1‐82Q and Httax1p‐97QP.^150^

Moreover, cancer stem cells (CSCs) are considered as the primary tumor initiator cells. It is becoming clear that TRIM proteins are involved in the regulation of CSCs. For example, TRIM16 directly regulates the degradation of Gli‐1 protein through the ubiquitin‐proteasome pathway, thereby inhibiting the CSC properties of BC cell populations, demonstrating it may contribute to a favorable prognosis for patients.^151^ It was shown that TRIM28 interacts with EZH2 and SWI/SNF to activate genes that promote mammosphere formation. High levels of TRIM28 are associated with the triple‐negative BC (TNBC) with high invasiveness and low survival rate, while the downregulation of TRIM28 reduces the ability of CSCs to self‐renew and leads to deceased expression of pluripotency and mesenchymal markers.^152^ Another study also implicated that long noncoding RNA (lncRNA)BMP/OP‐ResponsiveGene (BORG) promotes CSC phenotype and TNBC tumor initiation in mice through its ability to interact physically with the TRIM28.^153^ It was revealed that TRIM27 is a novel negative regulator of autophagy. TRIM27 cooperates with STK38L to inhibit ULK1‐mediated autophagy and contributes to tumorigenesis in BC.^38^, ^154^

### 3.2 Sustaining proliferation, resistance to apoptosis and metastasis of BC

The p53 signaling and TGF‐β signaling have attracted the most attention among the studies of TRIMs involved in BC proliferation and migration pathway. Therefore, we firstly focused on how the TRIM family proteins promote or inhibit BC progression through these two pathways. Then we discussed other TRIM proteins that were recently found to influence BC proliferation and migration.

#### 3.2.1 p53 hub

**FIGURE 2:**
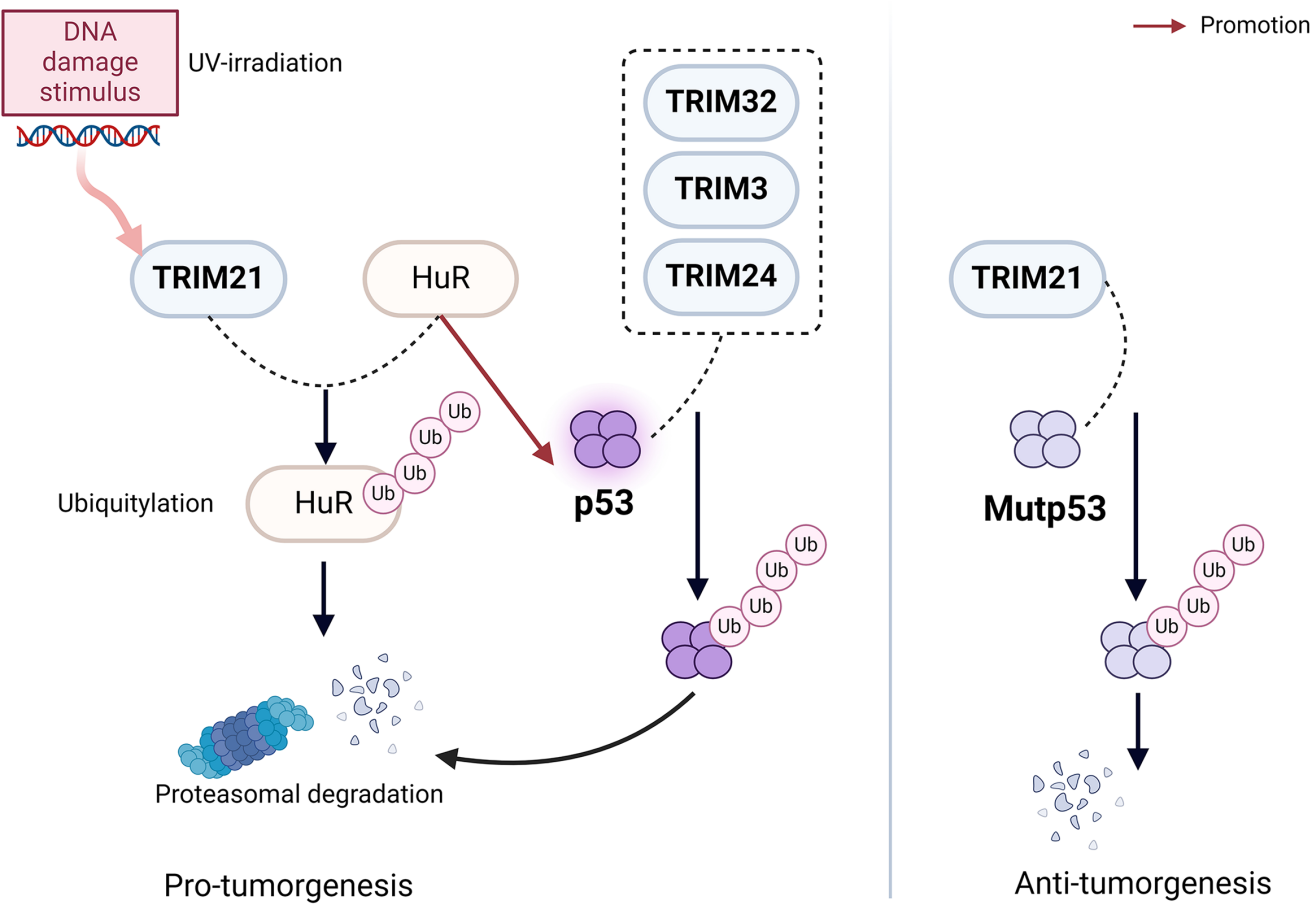
Diagram of TRIM family proteins interaction with p53 in BC.

Over the last several decades, researchers have developed an understanding of the fundamental role of p53 as a key tumor suppressor, and have been working on new approaches targeting p53 for treating p53‐mutated BC, such as PRIMA‐1, APR‐246, and COTI‐2, which could reactivate mutant p53 (mutp53) to convert it to a form with wild‐type (WT) properties.^155^, ^156^, ^157^ In addition to the oncoprotein MDM2, which is the primary E3 ubiquitin ligase for p53, an increasing amount of data suggests that p53 ubiquitination and degradation are more complex than once thought. TRIM proteins are involved in the multiple facets of this pathway (Figure 2). In Abhishek Guha's report, TRIM21 inhibits p53 protein synthesis by degrading the RNA‐binding protein HuR in response to stress, such as DNA damage.^14^, ^15^ Additionally, TRIM32,^158^ TRIM24,^159^ and TRIM3^23^ are endogenous inhibitors of p53 signal transduction as well, negatively regulating p53‐mediated apoptosis, cell cycle arrest, and senescence.

Mutp53 proteins often accumulate to extremely high levels in cancers to promote disease progression through the gain‐of‐function (GOF) mechanism.^160^, ^161^ In contrast to the cancer promoting features of TRIM21 mentioned above, another study by Juan Liu discovered that TRIM21 directly interacts with and degrades mutp53 but not WTp53, to suppress oncogenic GOF of mutp53.^162^ Mechanisms of these seemingly contradictory roles of TRIM21 in cancer remain elusive; however, we could partially explain the opposite results. Abhishek Guha's report focused on TRIM21 inhibiting p53 via HuR rather than the directly interaction. Further, in cancers carrying mutp53, mutp53 may play a more important role than WTp53.^162^, ^163^, ^164^ And the experimental methods in two studies also differ. In the study by Juan Liu, the inhibitory effect of TRIM21 was confirmed both in vitro (SK‐BR3 and HCC70) and in vivo, while the result of Abhishek Guha's study was only validated on BC cell lines (MCF7 and MDA‐MB‐231). Furthermore, TRIM31 can directly bind p53 inducing its K63‐linked ubiquitination, and meanwhile disrupt MDM2‐mediated K48‐linked ubiquitination of p53, both leading to the p53 stabilization and activation.^104^

#### 3.2.2 The TGF‐β signaling pathway

Transforming growth factor beta (TGF‐β) is being increasingly recognized as major regulators in epithelial tumor progression, notably as potent inducers of epithelial‐mesenchymal transition (EMT). Upon TGF‐β ligand activation and its binding to the receptor, the downstream target SMAD2/3 is phosphorylated and forms a heterodimeric complex with SMAD4, subsequently translocating to the nucleus and thereby transcriptionally activating EMT‐related genes.^165^

TRIM59 is verified to increase p‐SMAD2 expression and thus reinforce the activity of TGF‐β signaling.^166^ TRIM62 (annotated as DEAR1), which is expressed in normal ductal and glandular epithelial breast tissues, could finely regulate SMAD3 to inhibit TGFβ‐induced expression of EMT‐related genes in HMECs.^167^ TRIM62 is downregulated in transition to ductal carcinoma in situ (DCIS) and becomes an independent predictor of local recurrence‐free survival (RFS) in early‐onset BC.^168^, ^169^ Absent or downregulated TRIM62 could elevate the SMAD3 levels and its targets SNAIL1/2, which is the master transcriptional regulators of EMT, accelerating TGF‐β‐driven migration, invasion, and metastasis.^167^

**FIGURE 3:**
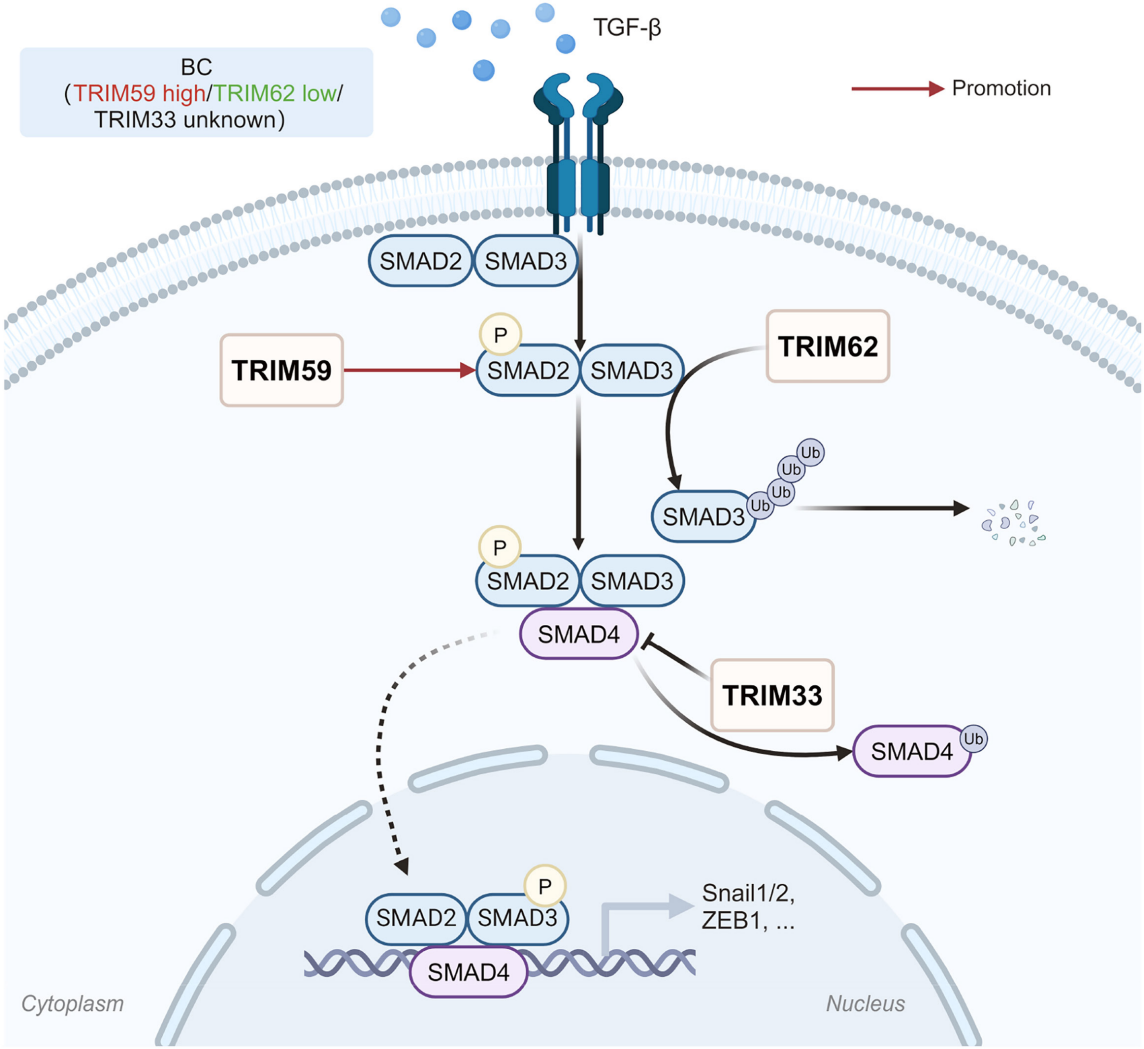
Diagram of TRIM family proteins involved in the TGF‐β signaling pathway in BC.

TRIM33, also known as TIF1γ, was discovered to antagonize SMAD4 during TGF‐β‐induced EMT in BC.^170^ Another previous study also proposed that TRIM33 could function as SMAD4 monoubiquitin ligase to promote its nuclear export and inhibit the formation of SMAD nuclear complexes.^171^ However, the prognostic significance of TRIM33 in BC patients is an area of many contradictions. One study revealed that TRIM33 expression in tissue samples carries a tendency towards poorer prognosis,^172^ while another prospective follow‐up study suggested those TRIM33 positive patients at plasma levels have a significantly improved overall survival (OS) compared with negative ones.^173^ The inconsistency between the two studies may be due to different means of detecting TRIM33 or different clinico‐pathological characteristics of the included patients. Though both enrolled operable BC patients, the former study mainly focused on French patients and Luminal subtype, whereas the latter one was conducted on Chinese and only less than 30% of the population was Luminal subtype. Further elaboration and validation are still required to establish TRIM33 as a biomarker in BC management (Figure 3).

#### 3.2.3 Other tumor positive regulators

Evidence thus far indicates that several other TRIM family members are also essential players of promoting BC progression. TRIM6 acts as a cancer promoter through degradation of STUB1 and provocation of YAP1 pathway.^174^ TRIM44,^175^ TRIM47,^176^ and TRIM63^177^ possess pro‐tumorigenic function by enhancing NF‐κB, PI3K/Akt, and Wnt/β‐catenin signaling, respectively. TRIM24, a chromatin reader, binds to the ER to activate tumor‐boosting estrogen‐dependent target genes.^145^ SUMOylation of TRIM24 promotes cell adhesion to extracellular matrix proteins, including fibronectin, laminin, and tenascin, thereby regulating cell adhesion.^178^ TRIM25 was proposed to be a potent pro‐metastatic transcription factor,^179^ while cumulative results provided compelling evidence that TRIM14,^180^ TRIM27,^181^ TRIM28,^182^ TRIM37,^114^ and TRIM59^183^ are also carcinogenic hallmarks in BC.

#### 3.2.4 Other tumor negative regulators

PDK1 was identified as a ubiquitination substrate of TRIM35. Overexpression of TRIM35 could halt tumor growth by inactivating AKT signaling through the increased degradation of PDK1.^184^ TRIM21 mediates ubiquitination degradation of SNAIL. BC models with TRIM21 R64Q mutation showed greatly increased infiltration into neighboring muscle fibers.^185^ TRIM29 antagonizes the activity of the TWIST1 oncogene,^186^ and it can abolish the upregulation of TWIST1 under hypoxic stress.^187^

### 3.3 Resistance to cancer therapies

The involvement of TRIM family in endocrine therapy resistance of BC has been extensively studied. TRIM27 could interfere with non‐TNBC cells and counteract the anticancer effects of tamoxifen (TAM) by mediating ubiquitination of p21.^188^ TRIM2 functions as a direct regulator to degrade an important drug‐induced proapoptotic protein, Bcl‐2 interacting mediator of cell death (Bim) in BC cells, taking part in the development of TAM‐resistance regulated by the GPER‐MAPK/ERK signaling.^189^ Both TRIM47^190^ and TRIM3^191^ were also reported to reduce the sensitization to endocrine therapy in BC.

A growing body of literature has solidified TRIM32 as a novel target in the radioresistance and chemoresistance of BC. Radiotherapy promotes the binding of CDK2 and TRIM32, resulting in increased CDK2‐dependent phosphorylation and nuclear translocation of TRIM32. Thereafter, nuclear TRIM32 enhances transcriptional activation of STAT3 and radioresistance in TNBC. As such, the research provided insights into therapeutic strategies of blocking radioresistance in BC based on CDK2/TRIM32/STAT3 signaling.^192^, ^193^ According to the study conducted by Zhao, TRIM32 downregulates p21/p27 and upregulates IAP family proteins to confer cisplatin resistance to BC and facilitate its growth through the NF‐κB signaling pathway.^194^

TRIM16 was identified to lead to an increase in retinoic acid (RA)‐responsive RARβ transcriptional activation and work as a pivotal regulator in the retinoid anticancer process.^195^ A recent study elucidated that TRIM47 overexpression induces ubiquitin ligase‐mediated proteasome turnover of BRCA1 and upregulate the sensitivity of TNBC cells to Olaparib, an inhibitor of poly‐(ADP‐ribose)‐polymerase (PARP).^196^ There was also evidence showing several other chemosensitivity mediators in BC, such as TRIM37^197^ and TRIM58.^198^

### 3.4 TRIMs in TIME

The complex interplay between cancer cells and the TIME influences the outcome of immunotherapy and other anticancer therapy.^199^ Abnormal accumulation of CD73 in cancer has been uncovered related to unfavorable survival in BC.^200^ TRIM21 governs CD73 destruction and decreases CD73‐catalyzed production of adenosine, thus enhancing CD8+ T cell function. The “TRIM21 high and CD73 low” subgroup of BC suggests a favorable immune profile.^201^ STAT6 is one of the vital transcription factors involved in macrophage M2 polarization. Preclinical studies showed that TRIM24 suppresses macrophage M2 polarization via ubiquitylation of the acetyltransferase CREB‐binding protein (CBP) at Lys119 and recruiting CBP to STAT6 achieving the acetylation of STAT6. These findings unveiled a finely tuned molecular mechanism for antitumor immune responses.^202^

Genomic analysis identified TRIM27 as a hallmark of tumors with high fibrosis and possible driver factor of fibrosis, which promotes the formation of an immunosuppressive TME through the activation of the TGF‐β signaling and represents an independent poor prognosis predictor of TNBC.^203^

### 3.5 Cell cycle and mitosis

Various recent reports have suggested the role of TRIM family members in cell cycle and mitosis. Studies revealed that TRIM37 blocks the formation of foci that comprise pericentriolar material—these foci are structures with a microtubule‐nucleating capacity, which is required for correct cell division in the absence of centrosomes.^204^, ^205^ Interestingly, researchers found an excess of TRIM37 leads to synthetic lethality with the polo‐like kinase 4 (PLK4) inhibitor, which provides a rationale for the use of centrosome targeting agents in treating tumors bearing TRIM37 overexpression. Notably, the TRIM37 is located in the chromosomal region 17q23, which is frequently amplified in several tumor types, including about 10% of BC cases.^206^ Similarly, TRIM16^207^ and TRIM62^208^ were reported to have the effect of cell cycle arrest induction. On the contrary, TRIM25^209^, ^210^ and TRIM39^211^ have been identified to drive cell cycle progression to promote BC growth. TRIM25 targets 14–3‐3 sigma, a negative cell cycle regulator that causes G2 arrest, for proteolysis and facilitates breast tumor progression.

### 3.6 Metabolic reprogramming

It has been widely documented that the initial cancer cells undergo metabolic reprogramming, such as the Warburg effect. High cellular glucose uptake has also been recognized as one of the hallmarks of cancer.^212^ Pathiraja et al observed that ectopic expression of TRIM24 in immortalized HMECs (TRIM24 iHMECs) would activate aerobic glycolysis as well as greatly increase glucose metabolism, and functionally promote the malignant biological behavior of BC.^213^ In contrast, TRIM35 overexpression restrains glycolysis and affects the Warburg effect negatively in BC cells. Pyruvate kinase M2 (PKM2) is the key enzyme in the Warburg effect, which catalyzes PEP to transfer phosphate groups to adenosine diphosphate to generate ATP. Mechanistic analysis indicated that TRIM35 regulates the ubiquitination modification of PKM2 and promote its alternation from dimers to tetramers.^214^

## 4 POTENTIAL OF TRIMs IN DIAGNOSIS AND DRUG DESIGNS

### 4.1 Alteration of TRIMs in BC and its prognostic roles

Alteration of TRIM protein expression is suggestive of BC development. TRIM13,^215^ TRIM21,^216^ TRIM35,^214^ TRIM58,^217^ and TRIM62^169^ are expressed at low levels in BC samples, whereas the expression of TRIM24,^218^ TRIM28,^219^, ^220^ TRIM39,^211^ TRIM44,^175^ TRIM59,^221^ and TRIM63^177^ maintains high. Analyzing the relationship between TRIM expression and clinic‐pathological features of patients, members such as TRIM24, TRIM28, and TRIM59 are revealed further upregulated in the subtype of TNBC which is usually more aggressive. Similarly, those lowly expressed in BC tissues like TRIM13, TRIM21, and TRIM62 are found associated with non‐TNBC. Meanwhile, dysregulation of these TRIM proteins could be poor prognostic factors for BC, which may help solve the challenges of constructing a practical and high‐quality model to predict the risk of death in BC patients. There are eight TRIM members revealed to be associated with OS, the gold standard primary endpoint to evaluate patients' outcome, while there are five molecules affecting disease‐free survival (DFS) or RFS (Table 3).

**TABLE 3:** Dysregulation of TRIM proteins and its related clinical characteristics.

Name	Tendency in BC	Prognosis indicator	Hazard ratio	*p* Value	Relative clinicopathological characteristics	References
TRIM13	Low	Metastatic RFS	0.8	0.0002	ER status, PR status, triple‐negative phenotype, and lymph node status	^215^
TRIM21	Low	OS	0.4	0.003	Tumor size, ER status, HER2 status, and clinical stage	^216^
TRIM31	Low	OS	—	0.04	Tumor size, Ki‐67 expression, clinical stage, histological grade, and lymph node status	^104^
TRIM35	Low	OS	0.8	0.018	—	^214^
TRIM58	Low	pCR	0.1	0.028	Tumor size	^217^
		OS	0.7	0.010		
TRIM62	Low	Local RFS	—	0.033	Family history of BC and triple‐negative phenotype	^169^
TRIM24	High	OS	5.0	0.025	Tumor stage, ER status, and worse molecular subtype	^218^
TRIM28	High	OS	3.1	0.048	Clinical stage, lymph node status, and worse molecular subtype	219, 220
		PFS	3.7	0.008		
		RFS	1.2	0.003	Age, tumor recurrence, p53 mutation status, PR status, and tumor stage	
TRIM39	High	DFS	2.3	0.049	Clinical stage, tumor size, and lymph node status	^211^
TRIM44	High	DFS	3.9	0.005	Nuclear grade and age	^175^
		OS	8.1	0.002		
TRIM59	High	OS	—	0.009	Clinical stage, lymph node status, and triple‐negative phenotype	^221^
TRIM63	High	—	—	—	Pathological differentiation and clinical stage	^177^

Abbreviations: pCR, pathological complete response; PFS, progression‐free survival.

Indeed, combining TRIM proteins with other proteins shows higher efficiency in clinical evaluation. Eight hundred and eighty‐nine cases of invasive breast adenocarcinoma from the TCGA cohort revealed a potential synergistic function of heterozygous loss of TRIM62 with SNAI2 alteration to significantly predict shortened overall survival (*p* = 0.023), while neither change alone significantly affects survival (*p* = 0.095 and 0.508, respectively).^167^ A Japanese invasive BC cohort demonstrated that double‐positive status for both the upstream regulator of the NF‐κB signaling pathway A20 and TRIM44 are more effective prognostic factors.^222^

### 4.2 The therapeutic potential of TRIM family

Targeting or activating TRIM proteins showed a promising therapeutic avenue to combat BC. Several theoretical bases have been developed and some of strategies targeting TRIM proteins have shown therapeutic potential in preclinical trials. As illustrated before, members like TRIM27, TRIM2, TRIM47, and TRIM3 can induce resistance to TAM in BC, suggesting the possible translation of these basic research findings into new endocrine therapeutics. Sorafenib, a standard treatment for HCC, could increase the sensitivity of TNBC cell lines to Olaparib by promoting TRIM21‐mediated ubiquitination degradation of BRCA1. Both in vitro and in vivo studies have proved the combination of Sorafenib and Olaparib exhibits a synthetic lethality effect, expanding their possibility of treating TNBC.^223^

Proteolysis‐targeting chimeric molecules (PROTACs) represent an emerging appealing technique that is receiving more and more attention for therapeutic intervention, providing new perspectives on the application of TRIM proteins. PROTACs are heterobifunctional molecules that contain three components: E3 ubiquitin ligase binding ligand, a linker, and the protein‐of‐interest (POI) binding moiety. Mechanistically, the proteasomal degradation of POI is initiated when PROTACs promote the POI and E3 to form ternary complex.^224^ For one thing, TRIMs can serve as a direct target for PROTACs. Considering that TRIM24 contains the PHD‐BRD dual epigenetic reader domain, small molecule inhibitors have been developed to target this module; however, the BC cell line MCF‐7 was found resistant to the tool compound in the concentration range tested.^225^ Recently, TRIM24‐PROTAC treatment was confirmed to successfully degrade TRIM24 and inhibit tumor growth in the TNBC patient‐derived xenograft models, and its potential could be further validated in early clinical trials in the future.^226^ For another, TRIMs could be mediators of PROTAC theoretically. PROTAC drugs might be designed to recruit TRIM proteins to specifically downregulate certain tumor POIs. The existing clinical and research gaps will gradually be filled to help eradicate BC in the future.

## 5 CONCLUSIONS

In the current review, we have systematically summarized the recent advances with respect to the role of TRIM proteins in BC. Ubiquitylation is the main manner in which TRIM family members control a wide range of proteins related to tumor occurrence and progression. As mentioned, many molecules have been identified to affect BC progression by ubiquitinating different substrates and thus by performing different functions (Table 1). For example, TRIM27 could directly polyubiquitinate ULK1 to negatively modulate autophagy as well as ubiquitinate p21 to interfere with chemoresistance, both leading to BC development. TRIM21 have also been widely studied, and its downstream ubiquitinated substrates include SNAIL, CD73, mutp53, finally exhibiting BC repressive functions. Interestingly, some members of the TRIM family were found to exert dual roles in the development of BC. However, if we compare those seemingly contradictory studies in detail, the experimental methods and subjects of the studies turn out different. To date, precise regulatory mechanisms of some TRIM proteins, such as the kind of polyubiquitin chains on the substrates remain to be determined. For instance, taking advantage of the genomic and transcriptomic data, researchers have discovered TRIM27 alterations in TNBCs with high fibrosis, but how it might drive the formation of fibrosis are still poorly understood. In summary, on the one hand, the functional significance of TRIMs makes them predictive biomarkers and potential drug targets. On the other, given that the complicated interconnections involving TRIMs in the tumor development, treatment strategies targeting TRIMs may still need further extensive research. It is believed that with the maturity of emerging modalities, TRIMs can provide an invaluable addition to the next‐generation anti‐BC therapeutics.

## AUTHOR CONTRIBUTIONS

**Jianing Cao:** Formal analysis (lead); writing – original draft (lead). **Mengdi Yang:** Funding acquisition (equal); visualization (equal); writing – review and editing (lead). **Duancheng Guo:** Validation (equal). **Zhonghua Tao:** Conceptualization (equal); project administration (equal); writing – review and editing (equal). **Xichun Hu:** Conceptualization (lead); funding acquisition (equal); supervision (lead); writing – review and editing (equal).

## FUNDING INFORMATION

This study was sponsored by the National Natural Science Foundation of China (82303398), Shanghai Sailing Program (22YF1408700), Excellent Physician Program of Fudan University Affiliated Cancer Hospital (ZYJH202109), National Science and Technology Major Project (2020ZX09201‐013, 2018ZX09301018‐002), Program for Shanghai Outstanding Academic Leader (LJRC2102, LJRC2102‐P), Shanghai Anticancer Association SOAR PROJECT (SACA‐AX202106).

## CONFLICT OF INTEREST STATEMENT

The authors declare no conflicts of interest.

## Data Availability

Not applicable.
